# Supplementary material for: The role of emotion regulation and intolerance to uncertainty on the relationship between fear of COVID-19 and distress
Source: Curr Psychol. 2022 Apr 25:1–12. Online ahead of print. doi: 10.1007/s12144-022-03071-5 (PMC9037968; doi:10.1007/s12144-022-03071-5)
Supplement: Supplementary file 1 — (DOCX 84 kb) [file 12144_2022_3071_MOESM1_ESM.docx]

**The role of emotion regulation and intolerance to uncertainty on the relationship between fear of COVID-19 and distress**

**Abstract**

The COVID-19 pandemic is increasing negative consequences on mental health around the globe. To date, research on what psychological factors could influence individuals’ distress is still scarce. The current study aims to test a multiple mediation model to examine the role of Intolerance of Uncertainty (IU) and emotional regulation (i.e., expressive suppression and cognitive reappraisal) as joint factors, which can increase understanding of psychological distress due to the COVID-19 outbreak. An online survey measuring fear of COVID-19, IU, emotional regulation, and distress was administered to 3863 Italian adults (females 73.3%; *M_age_* = 36.44; *SD* = 14.74) during lockdown. Results showed that IU partially mediated the effect of fear of COVID-19 on depression, anxiety and stress. Moreover, individuals with difficulties in managing the uncertainties due to their fear of COVID-19 may be at risk for heightened use of expressive suppression and depression. However, individuals with both higher IU and expressive suppression showed lower level of stress. High cognitive reappraisal has a mediational effect on the relationship between fear of COVID-19, IU, and lower distress. Findings suggest that IU and emotional regulation should be targeted for informing the development of tailored treatments to reduce the negative consequences of the outbreak.

*Keywords:* COVID-19; psychological distress; fear of COVID-19, intolerance of uncertainty, emotion regulation

**Introduction**

Since its outbreak in December 2019, the COVID-19 pandemic has caused noticeable psychological distress and negative emotional reactions (e.g., fear, uncertainty, anger) among the general population around the world (Mertens et al., 2020; Schimmenti et al., 2020). Measures of social distancing and quarantine aimed to limit the spread of the virus have had additional negative consequences on mental health Firstly, early evidence from Italy and China (Mazza et al., 2020; Wang et al., 2020) reported persistently elevated levels of stress, anxiety and depression, during the first outbreak, alongside symptoms of post-traumatic stress disorder, subsequently confirmed by other studies (Cao et al., 2020; Di Blasi et al., 2021). Recent reviews (Necho et al., 2021; Xiong et al., 2020) analyzing studies from western and eastern countries, revealed that the COVID-19 lockdown was associated with significant levels of psychological distress, which, in several cases, actually reach the threshold for clinical relevance. These findings suggest that the pandemic is having an overall negative effect on the population’s mental health. However, there is still limited research clarifying whether specific psychological factors (i.e., fear, uncertainty, emotion regulation) could play a key role in heightening or buffering psychological distress among the population.

Fear of COVID-19 has been highlighted as one of the factors, which may produce a negative impact on individuals’ distress; prior research evidenced the fact that the greater the fear of COVID-19, the higher the level of self-reported negative psychological consequences (Satici et al., 2020). Specifically, previous research suggested that fear of COVID-19 is associated with elevated health anxiety, psychological distress, loneliness and low life-satisfaction among non-clinical samples (Ahorsu et al., 2020; Lo Coco et al., 2021; Mertens et al., 2020; Satici et al., 2020). Moreover, high levels of worry and fear may be maladaptive and have a negative impact on the psychosocial well-being of the individual (Ornell et al., 2020).

Individuals with difficulties in handling the challenges experienced in response to the pandemic may be at greater risk of any heightened distress stemming from the COVID-19 outbreak. There is evidence that individuals who are younger, female, living without a partner, and those who have lost earnings, are at greater risk of negative health outcomes (Ellwardt & Präg, 2021). It is, therefore, important to ascertain whether there are any personal characteristics and regulatory strategies that may be heightening or buffering general distress among the population. In the current study, we have examined the mediating role of Intolerance of Uncertainty (IU) and emotion regulation strategies in the relationship between fear of COVID-19 and psychological distress during the first pandemic lockdown.

The proliferation of COVID-19 may present a special challenge for individuals with a low capacity to tolerate uncertainty. IU was defined as “an individual’s dispositional incapacity to endure the aversive response triggered by the perceived absence of salient, key, or sufficient information, and sustained by the associated perception of uncertainty” (Carleton, 2016, p. 31). The COVID-19 pandemic has brought about a high degree of uncertainty (Rettie & Daniels, 2020) and it is likely that individuals with a high fear of COVID-19 may experience difficult-to-tolerate, increased uncertainty due to the pandemic, with negative emotions and cognitions caused by their stressful experience. A recent study by Mertens and colleagues (2020) found that fear of COVID-19 was positively correlated with IU. Another study (Bakioğlu et al., 2020) suggested that IU and emotional distress may reinforce the negative effect of fear of COVID-19 on positive psychological states. Therefore, a contrasting relationship has also been suggested, where the inability to tolerate uncertainty during a pandemic may negatively predict psychological well-being, and this association may be mediated by fear of COVID-19 (Satici et al., 2020).

It has also been well established that emotion regulation processes play a crucial role in reducing or heightening negative emotions and distress (Aldao et al., 2010). Emotional regulation processes could be defined as the strategies used by an individual to modulate his/her emotional response to a given stimulus or situation, making it possible to modulate the intensity, duration and/or quality of the emotional experience and expression (Gross, 2014). In the process of emotion regulation, the *cognitive reappraisal* consists in modifying the cognitive meaning attributed to a situation, whereas the *expressive suppression* consists in inhibiting or reducing ongoing emotion-expressive behavior (Gross, 2001). There is evidence that the use of adaptive strategies such as cognitive reappraisal, problem solving and acceptance are associated with less anxiety and depressive symptoms, while the contrary holds for maladaptive strategies such as expressive suppression, avoidance and rumination (Aldao et al., 2010; Schäfer et al., 2017). Previous COVID-19 studies (Low et al., 2021; Xu et al., 2020) showed that expressive suppression increased the risk of poorer psychological health, whereas cognitive reappraisal showed weaker positive effects on psychological distress. Moreover, the use of expressive suppression resulted in increased levels of emotional distress in health-care workers who were at risk of exposure to COVID-19 (Garcia-Batista et al., 2020). Thus, it is likely that individuals with a high fear of COVID-19, experiencing uncertainty and negative emotions, will adopt dysfunctional emotion regulation strategies, which, in their turn, heighten psychological distress.

In the present study, we sought to provide a much-needed examination of the mental health impact of COVID-19 in a community-based sample and provide new insights regarding which individuals may be most vulnerable to psychological distress stemming from COVID-19 pandemic. Although there is evidence regarding the negative consequences that COVID-19 may have on mental health in terms of increased anxiety and depression symptomatology in both adult and adolescent populations (Benke et al., 2020; Meherali et al., 2021), there are important limitations in the literature as regards the restricted examination of the conjoint effects of IU and emotional dysregulation on an individual’s distress due to the COVID-19 outbreak. To our knowledge, only one study partially explored these relationships by examining the mediation role of IU, emotion regulation, and metacognitions in the relationship between fear of COVID-19 and health anxiety among family members of patients with COVID-19 (Akbari et al., 2021). The authors found that IU, expressive suppression, and metacognitions fully mediated the association between fear of COVID-19 and health anxiety. However, no previous research has investigated the link between fear of COVID-19 and psychological distress, through the mediating role of IU and emotion regulation in the general population. Thus, this study addresses this gap in knowledge, which may benefit our understanding of psychological suffering during the pandemic, whilst further fostering development of tailored psychological interventions for subsequent waves of COVID-19.

**Aim and** **Research Hypotheses**

Based on the literature reviewed above, we aim to examine the mediating role of IU and emotion regulation strategies in the link between fear of COVID-19 and psychological distress during the first COVID-19-related lockdown. The multiple mediation model configuring the relationship among variables is shown in Figure 1. We hypothesized that psychological distress (i.e., anxiety, depression and stress) during COVID-19 would be predicted by heightened levels of fear of COVID-19 (H1). We also hypothesized that the influence of fear of COVID-19 on an individual’s distress would be mediated by IU (H2), and, through a serial mediation model, via the conjoint effects of IU and emotion regulation (H3). Regarding H2, we expected that the introduction of IU would have a significant effect on psychological distress, over and above the effect of fear of COVID-19. As regards H3, according to literature on emotion regulation, we expected that cognitive reappraisal would foster a positive effect by reducing psychological distress, whereas expressive suppression would produce a negative effect by increasing the individual’s distress.

**Figure 1** Multiple mediation model (2 mediators).

*Note.* FCV-19S = Fear of COVID-19; IU = Intolerance of Uncertainty; reappraisal = ERQ reappraisal; suppression = ERQ suppression; distress = Psychological distress (DASS-21 stress, depression and anxiety); Indirect effect 1 (Ind1) = path *a_1_-b_1_*; Indirect effect 2 (Ind2) = path *a_2_-b_2_*; Indirect effect 3 (Ind3) = *a_1_-d_21_-b_2_*.

**Method**

**Participants**

The current study comprised 3863 Italian adults, mostly females (N = 2830; 73.3%); the participants’ ages ranged from 18 to 90 years (*M_age_* = 36.44; SD = 14.74). Only 15 respondents announced that they had been infected with COVID-19, 95 were uncertain about having been infected and given their limited representation (2.8%) in the overall sample, these subjects were excluded from the study; 9 did not answer this question. The remaining sample of 3768 subjects included individuals who live in different geographical areas of Italy, from northern to southern Italy, with educational levels ranging from primary to postgraduate specialization, and an employment status ranging from unemployment to retirement. Most of the participants reported that they spent their lockdown with one or more family members and adhered to requisite social distancing and shelter-in-place. Most of the participants had not presented any chronic pathology or disability during the previous year and about one fifth declared that they knew someone infected by the COVID-19 (see Table 1).

**Table 1** Sample characteristics (N = 3768)

|  | | **n** | **%** |
| --- | --- | --- | --- |
| Gender | Female | 2768 | 73.4 |
|  | Male | 1000 | 26.6 |
| Living Area | North-Italy | 1096 | 29.1 |
|  | Centre-Italy | 267 | 7.1 |
|  | South-Italy | 1481 | 39.3 |
|  | Not Reported | 924 | 24.5 |
| Educational level | Primary to High School | 1650 | 43.8 |
|  | Bachelor’s Degree | 705 | 18.7 |
|  | Master’s Degree or Postgraduate Specialization | 1413 | 37.5 |
| Occupational Status | Unoccupied | 182 | 4.8 |
|  | Unemployed | 244 | 6.4 |
|  | Student | 1145 | 30.3 |
|  | Employee | 1302 | 34.6 |
|  | Self-employed | 714 | 18.9 |
|  | Retired | 181 | 4.8 |
| Housing situation during lockdown | Alone | 268 | 7.1 |
|  | With one or more family members | 3218 | 85.4 |
|  | With other relatives or individuals external to the family | 192 | 5.1 |
| Health Status  (i.e., pathologies or disabilities) | Yes | 100 | 2.6 |
|  | No | 3664 | 96.8 |
|  | Not Reported | 4 | 0.0 |
| Knowing someone infected by COVID-19 | Yes | 793 | 21.6 |
|  | No | 2885 | 78.4 |
| Following the COVID-19 norms | Yes | 3722 | 98.8 |
|  | No | 46 | 1.2 |

**Procedure**

An online questionnaire was delivered through an e-mail link and text messages, and administered between April 7^th^ and 24^th,^ 2020, during the period of the COVID-19 quarantine. Before filling in the questionnaire, participants had to read and sign the informed consent. The study procedure was conducted in compliance with the Declaration of Helsinki and all subjects were informed that the data were anonymous and that they could omit any information they did not wish to give. Furthermore, they could withdraw from the study at any moment. The research was approved by the Ethical Committee of the University of Palermo (protocol code n. 3/2020—25 May 2020).

**Measures**

The questionnaire included sociodemographic information and comprised the following measures:

*Fear of COVID-19 Scale* (FCV-19S; Ahorsu et al., 2020) is a self-report questionnaire developed to assess the level of fear associated with COVID-19. The scale is based on a 5-point Likert scale, which investigates concerns about contagion, physiological reaction, states of nervousness and anxiety associated with both thinking about COVID-19 and searching for information about the virus through the media. Total scores range between 7 and 35, the higher the score the higher the fear of COVID-19. The internal consistency of the Italian version had been found to be adequate (α = .87) in a previous study (Soraci et al., 2020), just as in the present study, where alpha and omega were equal to .88.

*Depression Anxiety Stress Scale-21* (DASS-21; Bottesi et al., 2015; Lovibond & Lovibond, 1995) is a short 21-item self-assessment questionnaire based on a 5-point Likert scale that provides scores on three subscales: Stress, Depression and Anxiety. Higher scores correspond to a higher level of discomfort. The Italian version of DASS-21 (Bottesi et al., 2015) showed good internal consistency and good convergent and divergent validity, confirmatory factor analysis supported the goodness of the three-dimensions structure (anxiety, depression, and stress). In the present study, both *α* and *ω* were equal to .88 for Anxiety, equal to .90 for Depression, and respectively equal to .91 and .92 for the Stress subscale.

*Intolerance of Uncertainty Scale-Short Form* (IUS-12; Carleton et al., 2007; Lauriola et al., 2016) is a self-assessment questionnaire used to measure intolerance of uncertainty through 12 items with a 5-point Likert scale. The IUS provides a total score, with a higher score corresponding to a higher intolerance of uncertainty, resulting in difficulty in tolerating the negative affective state, triggered by a lack of information regarding a specific situation. The positive reliability of the Italian version (Luriola et al., 2016) was confirmed in the present study (*α*= .88, *ω*=.89) for the whole scale.

*Emotion Regulation Questionnaire* (ERQ; Balzarotti et al., 2010; Gross & John, 2003) is a self-assessment questionnaire based on 10 items measured on a 7-point Likert scale, which aims to evaluate the use of two specific strategies of emotion regulation: cognitive reappraisal and expressive suppression. For each of the two subscales, higher scores indicate a greater use of that regulation strategy. The Italian version of EQR (Balzarotti et al., 2010) showed that two-scale structure was confirmed and each of the two scales (Reappraisal and Suppression) showed good internal consistency. In the present study α and ω were .62 and .63 for cognitive reappraisal and .76 and .81 for the expressive suppression subscale.

**Data analysis**

Preliminary analyses were conducted to verify the normal distribution of the study variables. Variables are considered as highly skewed when indexes are below -2 or above +2 (George & Mallery, 2010). As regards kurtosis indexes, a normal distribution is defined as a mesokurtic distribution when kurtosis is equal to 3.

Comparisons between our sample and the normative population were performed by testing differences in average scores for psychological distress variables through a one-sample t-test.

The PROCESS macro in SPSS ([Hayes, 2013)](https://www.sciencedirect.com/science/article/pii/S0165032719309383?via%3Dihub#bib0017) was used for multiple mediation analyses, and a bootstrap method (N = 5000 times) was adopted to construct a 95% confidence interval for the significance testing of mediating effects. Process Model 4 was used to test a simple mediation model, with a single mediator (IU). Process Model 6 was then applied to test the simultaneous effect of two mediators (IU and emotional regulation). Therefore, in order to test alternative models, Process Model 6 was also applied to test the simultaneous effect of emotional regulation and FCV-19S as mediators of the relationship between IU and psychological distress. SPSS 22.0 was used to obtain descriptive statistics, correlation, and regression values.

**Results**

**Preliminary analyses**

Preliminary analysis of data distribution showed that there were no outliers and most of the variables highlighted an approximately symmetric distribution, with the exception of DASS-21 anxiety, which showed an acceptable skewness of 1.46. With regard to kurtosis indexes, most of the variables presented a platykurtic distribution, characterized by a higher and sharper central peak, and longer and fatter tails. Means for the general Italian population were compared with the current data, to establish whether there had been an increase in the individual’s distress during this period. The one-sample t-test showed that the means of our sample were significantly higher than those of the normative sample for the questionnaires (see Table 2). Specifically, our sample presented higher levels of depression (*d* = 0.57), stress (*d* = 0.44) and anxiety (*d* = 0.30). Given the high levels found, we decided *post-hoc* to compare the means of the DASS-21 scales with those reported for the clinical Italian population (Bottesi et al., 2015). Our sample differed from the clinical sample in the DASS-21 scales (see Table 2), as it presented lower levels of psychological anxiety (*d* = -0.37) and depression (*d* = -0.20), whereas no significant differences emerged for the stress scale (*d* = -0.0003). However, the average score for FCV-19S was found to be significantly lower when compared to scores reported in the validation study for the Italian version of FCV-19S (*d* = -0.27). Correlation among study variables are reported in Table 2.

**Table 2** Descriptive, Correlation and T-test one sample comparison with normative and clinical scores (N = 3768, *df* = 3767)

|  |  | 1 | 2 | 3 | 4 | 5 | 6 |
| --- | --- | --- | --- | --- | --- | --- | --- |
| 1 | FCV-19S | - |  |  |  |  |  |
| 2 | DASS-21 Anxiety | .50** | - |  |  |  |  |
| 3 | DASS-21 Depression | .31** | .65** | - |  |  |  |
| 4 | DASS-21 Stress | .39** | .69** | .78** | - |  |  |
| 5 | IU total | .38** | .41** | .49** | .47** | - |  |
| 6 | ERQ Reappr | -.04** | -.17** | -.26** | -.21** | -.16** | - |
| 7 | ERQ Suppr | .13** | .06** | .09** | 0.01 | .21** | .21** |
|  | Mean (SD) current sample | 15.20 (6.12) | 3.76 (4.52) | 6.60 (5.40) | 8.89 (5.64) |  |  |
|  | Mean normative sample | 16.86 | 2.4 | 3.5 | 6.4 |  |  |
|  | Mean clinical sample |  | 5.5 | 7.7 | 8.9 |  |  |
|  | t-value current *vs.* normative | -16.99** | 18.99** | 35.80** | 27.52** |  |  |
|  | t-value current *vs.* clinical |  | -23.47** | -12.53** | -0.22 |  |  |

*Note.* DASS-21 = Depression Anxiety Stress Scale-21; FCV-19S = Fear of COVID-19 Scale; **p<.001

Test of hypotheses

*H1 & H2: the role of the covariates and intolerance of uncertainty*

As expected for the H1, FCV-19S was associated significantly with DASS-21 anxiety (R^2^ = .23, β = .48, p<.01), depression (R^2^ = .10, β = .31, p<.01) and stress (R^2^ = .16, β = .39, p<.01), confirming its negative link to the individual’s mental health status during the lockdown. Several variables were taken into account as potential covariates: age, gender, occupation (dichotomized as occupied or not), impact of COVID-19 on economic status, living conditions (dichotomized as living alone or not), presence of chronic pathology during the previous year or disabled condition, and knowing someone infected by COVID-19. Among these variables, only age, gender, living conditions, impact of economic status and presence of chronic pathology during the previous year or disabled condition, showed significant effects and were included as covariates in the subsequent mediational models.

Regarding the second hypothesis of the study, a mediation model was run to establish whether IU mediated the relationship between FCV-19S and psychological distress over and above control variables. Results from PROCESS model 4 revealed significant effects of partial mediation for DASS-21 stress (effect = .89; BootLLCI-ULCI = .80-.99), depression (effect = 1.02; BootLLCI-ULCI = .92-1.12) and anxiety (effect = .51; BootLLCI-ULCI = .44-.58). Therefore, when IU was added to the model, the association of FCV-19S with stress decreased from 2.57 (β = .39) to 1.68 (β = .25), with depression dropped from 1.97 (β = .31) to 0.95 (β = .15) and with anxiety decreased slightly from 2.62 (β = .49) to 2.11 (β = .39), all remaining significant at p <.01. Regression coefficients showed that the higher the FCV-19S, the higher the IU and the higher the perception of distress in terms of all three DASS-21 scales. As expected, the introduction of IU explained a substantial part of variance psychological distress (ΔR^2^ = .16), and more than FCV-19S had done when the reverse model was tested by introducing FCV-19S after IU (ΔR^2^ = .02). In brief, these results indicated the presence of a partial mediation effect by IU, as FCV-19S continued to remain a significant predictor of all three DASS-21 scales. When covariates were introduced into the models, results remained substantially unmodified (results for models including covariates are shown in Online Resource 1).

*H3: The role of emotion regulation and intolerance to uncertainty as conjoint mediators*

Consistently with H3, we examined the mediation effect of both expressive suppression and cognitive reappraisal strategies on the relationships between FCV-19S, IU and psychological distress. The multiple mediation model (PROCESS model 6) is depicted in Figure 1 and the results are reported in Table 3.

The first model had DASS-21 stress as a dependent variable; the addition of cognitive reappraisal strategy to the model did not mediate the relationship between fear of COVID and stress (Ind2), whereas the indirect effect involving both mediators (Ind3) was significant. Therefore, the higher the FCV-19S, the higher the IU (a_1_= .38), and the lower cognitive reappraisal (d_21_= -.15) that, in turn, predicts a higher DASS-21 stress level (b_2_= -.11). We found a similar pattern of results when we added the expressive suppression to the model. Results showed that only the presence of both mediators has a significant effect (long path, Ind3): the higher the FCV-19S, then the higher the IU and higher expressive suppression may (d_21_= .20), in turn, predict lower DASS-21 stress levels (b_2_= -.07). Overall, negative *b_2_* coefficients showed that a higher use of both expressive suppression and cognitive reappraisal strategies were associated with lower stress.

**Table 3** Results of multiple mediation models; mediators: IU, Reappraisal and Suppression (N=3768)

|  | |  | **Path coefficients** | | | | | | | **Indirect effects** | |
| --- | --- | --- | --- | --- | --- | --- | --- | --- | --- | --- | --- |
|  | | | | | | | | | | | |
|  |  | | ***a_1_*** | ***a_2_*** | ***b_1_*** | ***b_2_*** | ***d_21_*** | ***c’*** |  | **effect** | **LLCI÷ULCI** |
| **DV: DASS-21 Stress** | | | | |  |  |  |  |  |  |  |
| Reappr |  | | .38* | -.01 | .32* | -.11* | -.15* | .25* |  |  |  |
|  | Ind1 | |  |  |  |  |  |  |  | .85^#^ | .76÷.95 |
|  | Ind2 | |  |  |  |  |  |  |  | .01 | -.02÷0.31 |
|  | Ind3 | |  |  |  |  |  |  |  | .05^#^ | .03÷.06 |
| Suppr |  | | .38* | .07* | .36* | -.07* | .20* | .26* |  |  |  |
|  | Ind1 | |  |  |  |  |  |  |  | .92^#^ | .76÷.97 |
|  | Ind2 | |  |  |  |  |  |  |  | -.03^#^ | -.05÷-.01 |
|  | Ind3 | |  |  |  |  |  |  |  | -.04^#^ | -.05÷-.02 |
|  |  | |  |  |  |  |  |  |  |  |  |

| **DV: DASS-21 Depression** | | | |  |  |  |  |  |  |  |
| --- | --- | --- | --- | --- | --- | --- | --- | --- | --- | --- |
| Reappr |  | .38* | .01 | .31* | -.17* | -.15* | .15* |  |  |  |
|  | Ind1 |  |  |  |  |  |  |  | .96^#^ | .87÷1.06 |
|  | Ind2 |  |  |  |  |  |  |  | .01 | -.03÷0.04 |
|  | Ind3 |  |  |  |  |  |  |  | .07^#^ | .04÷.08 |
| Suppr |  | .38* | .07* | .41* | .10* | .22* | .15* |  |  |  |
|  | Ind1 |  |  |  |  |  |  |  | 1.02^#^ | .92÷1.13 |
|  | Ind2 |  |  |  |  |  |  |  | .00 | -.01÷.02 |
|  | Ind3 |  |  |  |  |  |  |  | .03^#^ | .01÷.04 |
| **DV: DASS-21 Anxiety** | | | |  |  |  |  |  |  |  |
| Reappr |  | .38* | -.01 | .23* | -.09* | -.15* | .38* |  |  |  |
|  | Ind1 |  |  |  |  |  |  |  | .49^#^ | .42÷.56 |
|  | Ind2 |  |  |  |  |  |  |  | -.00 | -.01÷.02 |
|  | Ind3 |  |  |  |  |  |  |  | .03^#^ | .01÷.04 |
| Suppr |  | .38* | .08* | .20* | -.01 | .25* | .38* |  |  |  |
|  | Ind1 |  |  |  |  |  |  |  | .55^#^ | .44÷.59 |
|  | Ind2 |  |  |  |  |  |  |  | -.01 | -.02÷.01 |
|  | Ind3 |  |  |  |  |  |  |  | -.01 | -.02÷.01 |

*Note.* IU = Intolerance of Uncertainty; DASS-21 = Depression Anxiety Stress Scale-21; Reappr = Reappraisal; Suppr = Suppression; Ind = Indirect; *p < 0.01; ^#^CI does not contain zero

Regarding the model including DASS-21 anxiety as a dependent variable, the significant path Ind3 showed a conjoint mediation effect of cognitive reappraisal and IU on the link between FCV-19S and DASS-21 anxiety. Consistently with H3, the cognitive reappraisal strategy was negatively associated with DASS-21 anxiety (b_2_= -.09), showing that a higher utilization of reappraisal reduced anxiety. On the other hand, the addition of expressive suppression to the model did not result in any significant mediation in either path, Ind2 or Ind3.

Finally, when DASS-21 depression was considered as the dependent variable in the model, the cognitive reappraisal strategy confirmed its conjoint mediating role, as demonstrated by the results for the long, indirect path (Ind3). Higher levels of cognitive reappraisal were associated with lower levels of depression (b_2_= -.17). No mediation effect of cognitive reappraisal was found for the short path (Ind2). The expressive suppression strategy did not show a significant effect of mediation in the relationship between FCV-19S and DASS-21 depression (Ind2), whereas in the long path (Ind3) we found that the higher the level of FCV-19S, the higher the level of IU, and the higher the level of expressive suppression (d_21_= .22), in turn, predicted a higher level of DASS-21 depression (b_2_= .10). The results described above remained substantially unmodified after the introduction of covariates in the models (See Online Resource 2).

Considering the cross-sectional nature of the study, alternative models were tested in order to verify the simultaneous effect of FCV-19S and emotion regulation mediators in the relationship between UI and psychological distress.

The procedure followed was the same; results summarized in Table 4 showed that in no case was the portion of variability explained (R^2^) as being higher or similar to the variability explained by the hypothesized models that consider FCV-19S as the independent variable. The considerations reported here are also corroborated by the indirect effect (Ind3). Indeed, in the alternative models, Ind3 resulted not significant or weaker than the corresponding Ind3 effect in the hypothesized models. Only in one case, when DASS-21 anxiety was considered as outcome and suppression as second mediator, was Ind3 in the alternative model found to be higher than the respective Ind3 in the proposed model (see Online Resource 3).

**Table 4** Comparison, based on R-sq value, between the chosen model and the alternative model with UI as independent variable (N=3768)

|  | **Y** | **X** | **M1** | **M2** | **R^2^** |
| --- | --- | --- | --- | --- | --- |
| Hypothesized model  (H3) | DASS-21 Depression | FCV-19S | IU | Reappr | 0.335 |
|  | DASS-21 Depression | FCV-19S | IU | Suppr | 0.310 |
|  | DASS-21 Anxiety | FCV-19S | IU | Reappr | 0.340 |
|  | DASS-21 Anxiety | FCV-19S | IU | Suppr | 0.334 |
|  | DASS-21 Stress | FCV-19S | IU | Reappr | 0.380 |
|  | DASS-21 Stress | FCV-19S | IU | Suppr | 0.372 |
| Reverse model | DASS-21 Depression | IU | FCV-19S | Reappr | 0.296 |
|  | DASS-21 Depression | IU | FCV-19S | Suppr | 0.262 |
|  | DASS-21 Anxiety | IU | FCV-19S | Reappr | 0.303 |
|  | DASS-21 Anxiety | IU | FCV-19S | Suppr | 0.293 |
|  | DASS-21 Stress | IU | FCV-19S | Reappr | 0.297 |
|  | DASS-21 Stress | IU | FCV-19S | Suppr | 0.288 |

*Note.* Y = dependent variable; X = independent variable; M1 = first mediator; M2 = second mediator; R^2^ = coefficient of determination; Reappr = Reappraisal; Suppr = Suppression; IU = Intolerance of Uncertainty, DASS-21 = Depression Anxiety Stress Scale-21; FCV-19S = Fear of COVID-19

**Discussion**

From March 11 until May 3, 2020, the Italian government adopted extraordinary restrictive measures to contain the risk of contagion. During the lockdown, citizens were not allowed to enter, exit, or move around the country or from their cities, unless in case of proven necessity, or for work or health reasons. Schools and universities and all the non-essential activities were closed, and public gatherings were forbidden. Despite previous studies evidenced the detrimental psychological repercussions of social isolation due to the COVID-19 lockdown (Roma et al., 2020; Preti et al., 2021), several aspects of the impact of lockdown restrictive measures on mental health remain still unexplored. The present study sought to investigate the impact of COVID-19 outbreak on mental health in a community-based sample, by examining the role of IU and emotion regulation. The examination of psychological factors that may influence the levels of the individual’s distress during the pandemic were key research priorities (Holmes et al., 2020). Our findings showed heightened levels of anxiety and depression among participants during the first lockdown (medium effect size), although the stress levels were not significantly different from those reported from clinical populations. Overall, these findings are in line with those from previous studies, which evidenced that lockdown was a stressful experience, with high levels of psychological distress in community samples (Cao et al., 2020; Germani et al., 2020; Wang et al., 2020), by providing information about the negative impact that emergency alert and restriction measures had on the individual’s psychosocial well-being (Ahorsu et al., 2020).

Regarding the main study hypotheses, fear of COVID-19 was associated significantly with stress, anxiety and depression, consistently with previous studies (Fitzpatrick et al., 2020; Mertens et al., 2020), which showed that individuals reporting a greater fear of COVID-19 reported greater mental health difficulties. In particular, in this study, DASS-21 anxiety resulted strongly associated with fear of COVID-19, thus supporting the idea of a partial overlapping between these two aspects of the individual’s negative reactions. These relationships may partly be explained by a self-reinforcing circle in which, in turn, anxiety and fear increase negative feelings, which then may influence the individual’s ability to make rational decisions and consequently result in risk-taking behavior (Schimmenti et al., 2020).

Moreover, the findings of the current study suggest that fear of COVID-19 may predict an individual’s psychological distress both directly and through the role of heightened IU. Our results revealed the partial mediating role of IU in increasing the effect of fear of COVID-19 on stress, depression and anxiety. These results are in line with findings from previous research (Bakioğlu et al., 2020; Satici et al., 2020), highlighting the fact that the effects of fear of COVID-19 on psychological distress might be mediated by the individual’s tendency to tolerate the uncertainty intrinsic to the current pandemic situation. This finding is also in line with the model of uncertainty distress by Freeston and colleagues (2020), which posits that the more the individual is IU the more he/she perceives uncertainty as aversive, thus enhancing the perception of distress. Indeed, the current findings seems to support the role of IU in affecting psychological adjustment to potentially threatening situations (Freeston et al., 2020; Ouellet et al., 2019).

Previous research showed that the current pandemic and the related lockdown may have severe consequences on psychological health, as they bring about feelings of fear and uncertainty; these might then further exacerbate worry and negative emotions associated with perceived risk of COVID-19 infection and mortality, thus contributing to increased psychological distress and health anxiety (Akbari et al., 2021; Tull et al., 2020Moreover, previous research demonstrated that protracted, high-stress levels, irritability and agitation, with consequent difficulties in relaxing, may have significant negative consequences for people’s mental health, increasing vulnerability to post-traumatic stress disorder (Sun et al., 2020). Not surprisingly, the ability to tolerate uncertainty emerged as a key factor during the pandemic; this finding may suggest the importance of adopting all possible strategies to reduce uncertainty and to teach people to acquire psychological resources to tolerate uncertainty and downregulate negative emotions.

The current study adds to the literature on IU regarding the role of emotion regulation as an individual factor that can protect or exacerbate mental health during the COVID-19 outbreak. Multiple mediational models were used to investigate the effect of emotion regulation strategies on the relationships between fear of COVID-19, IU and psychological distress. Our findings showed that the cognitive reappraisal strategy has a mediational effect on the relationship between fear of COVID-19, IU, and psychological distress. Specifically, the higher the fear of COVID-19, the higher the IU, and the lower the reappraisal, which in turn predicts heightened anxiety and depression. Therefore, our findings suggest that a high application of cognitive reappraisal may be associated with lower psychological distress, thus confirming its widely accepted role as protective factor for psychological health (Gross & John, 2003; Schäfer et al., 2017). These findings are in line with a prior study by Xu and colleagues (2020) highlighting the fact that cognitive reappraisal negatively moderated the association between perceived stress and anxiety symptoms in a sample of COVID-19 isolated people.

Regarding the role of expressive suppression, we found a different pattern of results. Consistently with our hypothesis, higher levels of fear of COVID-19 and IU were associated with higher suppression, in turn resulting in heightened depression. This finding seems to be in line with the majority of cross-sectional studies with non-clinical samples (Dryman & Heimberg, 2018), which reported that expressive suppression may increase the level of depression, thus working as a maladaptive strategy, which ends up increasing the risk of a reduced mental health status (Low et al., 2021). Therefore, individuals with difficulties in managing the uncertainties experienced in response to fear of COVID-19 may be at greater risk because of heightened expressive suppression and depressive levels. However, contrary to expectations, expressive suppression was found to lessen the level of stress. This finding seems counterintuitive in relation to the literature on emotion regulation, which supports the fact that expressive suppression can play an important role in increasing stress-related symptoms (Schäfer et al., 2017). Moreover, previous research in the context of the COVID-19 pandemic showed that healthcare workers exposed to patients with COVID-19 tend to predominantly employ expressive suppression, thus increasing their perceived stress (Garcia-Batista et al., 2020). A possible explanation could be that, in times of a rapidly deteriorating situation, the employment of cognitive reappraisal may be an unworkable option and suppression the best choice, as suggested by Gross and John (2003). Considering the stressful nature and the rapid spread of the pandemic in 2020, we might speculate that the use of an emotional suppression strategy may be perceived as an adaptive reaction that buffers emotional expression to allow a subsequent, less emotional, reassessment of the situation (Tyra et al., 2021). Moreover, it should be noted that within the unpredictable context of the first wave of the pandemic, the higher reported levels of stress symptoms in our sample (which meet the threshold for clinical relevance) made it hard for individuals to subsequently change and reduce the intensity of their emotional responses through reinterpretation of the situation.

Moreover, the results of the mediational model suggested that individuals with low levels of fear of COVID-19 and IU are more likely to adopt cognitive reappraisal strategies, which might trigger a virtuous circle, improving the individual’s psychological health. On the contrary, individuals who experienced higher levels of IU and fear of COVID-19 showed a more extensive use of suppression rather than reappraisal in regulating their negative emotions. This finding may illustrate a feedback reinforcement mechanism in which an elevated fear of COVID-19 and IU may intensify expressive suppression in a vicious circle, reducing stress whilst, at the same time, heightening depression. Finally, results from the current study showed that a higher use of both expressive suppression and cognitive reappraisal strategies were associated with lower stress. This result partially reflects those of Tyra et al. (2021) showing a significant interaction of suppression by reappraisal for the prediction of distress. Specifically, simple effects evidenced that expressive suppression was negatively associated with acute stress only when cognitive reappraisal levels were high. These findings may support the hypothesis that the flexible combination of both cognitive reappraisal and expressive suppression strategies may represent adaptive emotional coping to dampen the initial emotional response to unpredictable events such as the COVID-19 pandemic. Further studies are needed to examine in depth the interplay between the use of reappraisal and suppression in response to stressful events and verify and extend these results.

The findings of the current study may have some relevant clinical implications, by indicating that emotion regulation difficulties should be considered to ameliorate individual’s distress following uncertain events linked to the pandemic. More specifically, mental health professionals should work at improving cognitive reappraisal strategies that can foster positive effects on depression and anxiety. Moreover, prior study indicated that early identification of individuals’ acute stress reactions following a potentially traumatic event may be useful for identifying those who may benefit from early intervention (Bryant et al., 2014). Along this line, earlier studies (Rettie et al., 2020; Tull et al., 2020) pointed out that reappraisal training and mindfulness-based behavioral interventions could provide psychological support to those individuals who have greater difficulty coping with uncertainty, by helping them to downregulate negative emotion and increasing their sense of control.

**Limitations**

While our study has a number of strengths, including a large sample size and testing with a multiple mediation model, several limitations should also be noted. First, the study is significantly limited in terms of revealing causal interferences, because it is a cross-sectional examination; longitudinal studies are needed to confirm the causal order of the associations between our study variables at different stages of the COVID-19 restrictions. Second, the study only included measures of psychological distress and did not examine other specific constructs such as COVID-19-related distress (Taylor et al., 2020), which have proven to comprise specific pandemic-related threats and traumatic stress symptoms with regard to the pandemic. Thus, further replications considering various psychopathological constructs are warranted. Moreover, recruitment occurred within a community context using a snowball sample. Although this provides important information regarding the effects that the COVID-19 pandemic and the related restrictive measures produced on the psychological health of non-clinical populations, it would be important to repeat this study with more representative samples. Finally, it is worth noting that, though acceptable, the internal consistency was low for the cognitive reappraisal subscale and that, in the current study, the fear of COVID-19 scores resulted lower (with a small effect size) than those previously found in an Italian sample (Soraci et al., 2020). However, there is evidence that fear of COVID-19 is not uniformly distributed across the population regards different regions (Fitzpatrick et al., 2020), and further national studies are necessary to determine the level of fear of COVID-19 among adults in different countries.

**Conclusions**

This study may shed light on the possible mechanisms underlying fear of COVID-19 and psychological distress, by considering the conjoint mediator role of IU and emotion regulation strategies. The findings highlight the fact that individuals with an elevated fear of COVID-19 and experiencing uncertainty may adopt dysfunctional emotion regulation strategies, which in turn might heighten psychological distress.

As such, this study evidences the importance of targeting modifiable factors for preventing and managing psychological health during the second wave of COVID-19.

**Supplementary Information**

**Online Resource 1: OUTPUTS OF MODERATION ANALYSES FOR THE HYPOTHESIZED MODEL;**

**Online Resource 2: OUTPUTS OF MODERATION ANALYSES FOR THE REVERSE MODEL;**

**Data policy**: The datasets generated during and/or analysed during the current study are available from the corresponding author on reasonable request

**References**

Ahorsu, D. K., Lin, C. Y., Imani, V., Saffari, M., Griffiths, M. D., & Pakpour, A. H. (2020). The fear of COVID-19 scale: development and initial validation. *International Journal of Mental Health and Addiction,* 1–9. <https://doi>.org/[10.1007/s11469-020-00270-8](https://doi.org/10.1007/s11469-020-00270-8)

Akbari, M., Spada, M. M., Nikčević, A. V., & Zamani, E. (2021). The relationship between fear of COVID‐19 and health anxiety among families with COVID‐19 infected: The mediating role of metacognitions, intolerance of uncertainty, and emotion regulation. *Clinical Psychology & Psychotherapy*. <https://doi>.org/ 10.1002/cpp.2628.

Aldao, A., Nolen-Hoeksema, S., & Schweizer, S. (2010). Emotion-regulation strategies across psychopathology: A meta-analytic review. *Clinical Psychology Review, 30*(2): 217–237. <https://doi>.org/[10.1016/j.cpr.2009.11.004](https://doi.org/10.1016/j.cpr.2009.11.004)

Bakioğlu, F., Korkmaz, O., & Ercan, H. (2020). Fear of COVID-19 and Positivity: Mediating Role of Intolerance of Uncertainty, Depression, Anxiety, and Stress. *International Journal of Mental Health and Addiction*, 1–14. <https://doi>.org/10.1007/s11469-020-00331-y

Balzarotti, S., John, O. P., & Gross, J. J. (2010). An Italian adaptation of the emotion regulation questionnaire. *European Journal of Psychological Assessment, 26*(1), 61–67. <https://doi>.org/[10.1027/1015-5759/a000009](https://doi.org/10.1027/1015-5759/a000009)

Benke, C., Autenrieth, L. K., Asselmann, E., & Pané-Farré, C. A. (2020). Lockdown, quarantine measures, and social distancing: Associations with depression, anxiety and distress at the beginning of the COVID-19 pandemic among adults from Germany. *Psychiatry Research, 293,* 113462. https://doi.org/10.1016/j.psychres.2020.113462

Bottesi, G., Ghisi, M., Altoè, G., Conforti, E., Melli, G., & Sica, C. (2015). The Italian version of the Depression Anxiety Stress Scales-21: Factor structure and psychometric properties on community and clinical samples. *Comprehensive Psychiatry*, *60*, 170–181. <https://doi>.org/[10.1016/j.comppsych.2015.04.005](http://dx.doi.org/10.1016/j.comppsych.2015.04.005)

Bryant, R. A., Creamer, M., O’Donnell, M., Silove, D., McFarlane, A. C., & Forbes, D. (2014). A comparison of the capacity of DSM-IV and DSM-5 acute stress disorder definitions to predict posttraumatic stress disorder and related disorders. *The Journal of Clinical Psychiatry*, *76*, 391–397. <https://doi>.org/10.4088/jcp.13m08731

Cao, W., Fang, Z., Hou, G., Han, M., Xu, X., Dong, J., & Zheng, J. (2020). The psychological impact of the COVID-19 epidemic on college students in China. *Psychiatry Research*, 112934. <https://doi>.org/[10.1016/j.psychres.2020.112934](https://doi.org/10.1016/j.psychres.2020.112934)

Carleton, R. N. (2016). Into the unknown: a review and synthesis of contemporary models involving uncertainty. *Journal of Anxiety Disorders*, *39*, 30–43. <https://doi>.org/10.1016/j.janxdis.2016.02.007

Carleton, R. N., Norton, P. J., & Asmundson, G. J. G. (2007). Fearing the unknown: A short version of the Intolerance of Uncertainty Scale. *Journal of Anxiety Disorders*, *21*, 105–117. <https://doi>.org/10.1016/j.janxdis.2006.03.014

Di Blasi, M., Gullo, S., Mancinelli, E., Freda, M. F., Esposito, G., Gelo, O. C. G., … & Lo Coco, G. L. (2021). Psychological distress associated with the COVID-19 lockdown: A two-wave network analysis. *Journal of affective disorders*, *284*, 18–26. https://doi.org/10.1016/j.jad.2021.02.016

Dryman, M. T., & Heimberg, R. G. (2018). Emotion regulation in social anxiety and depression: A systematic review of expressive suppression and cognitive reappraisal. *Clinical Psychology Review*, *65*, 17–42. https://doi.org/10.1016/j.cpr.2018.07.004

Ellwardt, L., & Präg, P. (2021). Heterogeneous mental health development during the COVID-19 pandemic in the United Kingdom. *Scientific Reports*, *11*, 15958. https://doi.org/10.1038/s41598-021-95490-w

Fitzpatrick, K. M., Drawve, G., & Harris, C. (2020). Facing new fears during the COVID-19 pandemic: The state of America’s mental health. *Journal of Anxiety Disorders*, *75*, 102291. https://doi.org/10.1016/j.janxdis.2020.102291

Freeston, M., Tiplady, A., Mawn, L., Bottesi, G., & Thwaites, S. (2020). Towards a model of uncertainty distress in the context of Coronavirus (COVID-19). *The Cognitive Behaviour Therapist*, 1–32. https://doi.org/10.1017/S1754470X2000029X

García-Batista ZE, Guerra-Peña K, Nouri Kandany V, Marte MI, Garrido LE, Cantisano-Guzmán LM, et al. (2021) COVID-19 pandemic and health worker stress: The mediating effect of emotional regulation. *PLoS ONE 16*(11): e0259013. https://doi.org/10.1371/journal.pone.0259013

George, D., & Mallery, M. (2010). SPSS for Windows Step by Step: A Simple Guide and Reference, 17.0 update (10a ed.) Boston: Pearson.

Germani, A., Buratta, L., Delvecchio, E., & Mazzeschi, C. (2020). Emerging Adults and COVID-19: The Role of Individualism-Collectivism on Perceived Risks and Psychological Maladjustment. *International Journal of Environmental Research and Public Health*, *17*(10), 3497. https://doi.org/[10.3390/ijerph17103497](https://doi.org/10.3390/ijerph17103497)

Gross, J. J. (2001). Emotion regulation in adulthood: Timing is everything. *Current Directions in Psychological Science, 10,* 214–219. https://doi.org/10.1111/1467-8721.00152

Gross, J. J. (Ed.). (2014). *Handbook of Emotion Regulation* (2nd ed.). New York: Guilford Publications.

Gross, J. J., & John, O. P. (2003). Individual differences in two emotion regulation processes: Implications for affect, relationships, and well-being. *Journal of Personality and Social Psychology*, *85*(2), 348–362. https://doi.org/[10.1037/0022-3514.85.2.348](https://doi.org/10.1037/0022-3514.85.2.348)

Hayes, A. F. (2013). *Methodology in the social sciences.Introduction to mediation, moderation, and conditional process analysis: A regression-based approach*. New York: Guilford Publications.

Holmes, E. A., O’Connor, R. C., Perry, V. H., Tracey, I., Wessely, S.,Arseneault, L., … & Bullmore, E. (2020). Multidisciplinary researchpriorities for the COVID-19 pandemic: A call for action for mentalhealth science. *The Lancet Psychiatry,* *7*, 547–560. https://doi.org/10.1016/S2215-0366(20)30168-1

Lauriola, M., Mosca, O., & Carleton, R. N. (2016). Hierarchical factor structure of the intolerance of uncertainty scale short form (IUS-12) in the Italian version. *TPM: Testing, Psychometrics, Methodology in Applied Psychology*, *23*(3), 377–394. https://doi.org/10.4473/TPM23.3.8

Lo Coco, G., Gentile, A., Bosnar, K., Milovanovic, I., Bianco, A., Drid, P., & Pišot, S. (2021). A Cross-Country Examination on the Fear of COVID-19 and the Sense of Loneliness during the First Wave of COVID-19 Outbreak. *International Journal of Environmental Research and Public Health, 18*, 2586. https://doi.org/10.3390/ijerph18052586

Lovibond, P. F., & Lovibond, S. H. (1995). The structure of negative emotional states: Comparison of the Depression Anxiety Stress Scales (DASS) with the Beck Depression and Anxiety Inventories*. Behaviour Research and Therapy, 33*, 335–342. https://doi.org/10.1016/0005-7967(94)00075-u

Low, R. S. T., Overall, N. C., Chang, V. T., Henderson, A. M. E., & Sibley, C. G. (2021). Emotion regulation and psychological and physical health during a nationwide COVID-19 lockdown. *Emotion,* *21*(8), 1671–1690. https://doi.org/10.1037/emo0001046

Mazza, C., Ricci, E., Biondi, S., Colasanti, M., Ferracuti, S., Napoli, C., & Roma, P. (2020). A nationwide survey of psychological distress among Italian people during the COVID-19 pandemic: immediate psychological responses and associated factors. *International journal of environmental research and public health*, *17*(9), 3165. https://doi.org/10.3390/ijerph17093165

Meherali, S., Punjani, N., Louie-Poon, S., Abdul Rahim, K., Das, J. K., Salam, R. A., & Lassi, Z. S. (2021). Mental health of children and adolescents amidst COVID-19 and past pandemics: a rapid systematic review. *International journal of environmental research and public health, 18*(7), 3432. https://doi.org/10.3390/ijerph18073432

Mertens, G., Gerritsen, L., Duijndam, S., Salemink, E., & Engelhard, I. M. (2020). Fear of the coronavirus (COVID-19): Predictors in an online study conducted in March 2020. *Journal of Anxiety Disorders*, *74*, 102258. https://doi.org/[10.1016/j.janxdis.2020.102258](https://doi.org/10.1016/j.janxdis.2020.102258)

Necho, M., Tsehay, M., Birkie, M., Biset, G., & Tadesse, E. (2021). Prevalence of anxiety, depression, and psychological distress among the general population during the COVID-19 pandemic: A systematic review and meta-analysis. *International Journal of Social Psychiatry*, *67*(7), 892–906. https://doi.org/10.1177/00207640211003121

Ornell, F., Schuch, J. B., Sordi, A. O., & Kessler, F. H. P. (2020). “Pandemic fear” and COVID-19: mental health burden and strategies. *Brazilian Journal of Psychiatry*, *42*(3), 232–235. https://doi.org/10.1590/ 1516-4446-2020-0008

Ouellet, C., Langlois, F., Provencher, M., & Gosselin, P. (2019). Intolerance of uncertainty and difficulties in emotion regulation: Proposal for an integrative model of generalized anxiety disorder. *European Review of Applied Psychology-revue Europeenne De Psychologie Appliquee*, *69*, 9–18. https://doi.org/10.1016/j.erap.2019.01.001

Preti, E., Di Pierro, R., Perego, G., Bottini, M., Casini, E., Ierardi, E., ... & Di Mattei, V. (2021). Short-term psychological consequences of the COVID-19 pandemic: Results of the first wave of an ecological daily study in the Italian population. *Psychiatry Research,* *305*, 114206. https://doi.org/10.1016/j.psychres.2021.114206

Rettie, H., & Daniels, J. (2020). Coping and tolerance of uncertainty: Predictors and mediators of mental health during the COVID-19 pandemic. *American Psychologist, 76*(3), 427–437. https://doi.org/10.1037/amp0000710

Roma P, Monaro M, Colasanti M, Ricci E, Biondi S, Di Domenico A, Verrocchio MC, Napoli C, Ferracuti S, Mazza C. (2020). A 2-Month Follow-Up Study of Psychological Distress among Italian People during the COVID-19 Lockdown. *International Journal of Environmental Research and Public Health.* *17(*21):8180. https://doi.org/10.3390/ijerph17218180

Satici, B., Gocet-Tekin, E., Deniz, M. E., & Satici, S. A. (2020). Adaptation of the Fear of COVID-19 Scale: Its association with psychological distress and life satisfaction in Turkey. *International* *Journal of Mental Health and Addiction*, 1–9. https://doi.org/10.1007/s11469-020-00294-0

Satici, B., Saricali, M., Satici, S. A., & Griffiths, M. D. (2020). Intolerance of uncertainty and mental wellbeing: Serial mediation by rumination and fear of COVID-19. *International Journal of Mental Health and Addiction,* 1–12. https://doi.org/10.1007/s11469-020-00305-0

Schäfer, J. Ö., Naumann, E., Holmes, E. A., Tuschen-Caffier, B., & Samson, A. C. (2017). Emotion regulation strategies in depressive and anxiety symptoms in youth: A meta-analytic review. *Journal of Youth and Adolescence*, *46*(2), 261–276. https://doi.org/10.1007/s10964-016-0585-0

Schimmenti, A., Billieux, J., & Starcevic, V. (2020). The four horsemen of fear: An integrated model of understanding fear experiences during the COVID-19 pandemic. *Clinical Neuropsychiatry: Journal of Treatment Evaluation, 17*(2), 41–45. https://doi.org/10.36131/CN20200202

Soraci, P., Ferrari, A., Abbiati, F. A., Del Fante, E., De Pace, R., Urso, A., & Griffiths, M. D. (2020). Validation and psychometric evaluation of the Italian version of the Fear of COVID-19 Scale. *International Journal of Mental Health and Addiction*, 1–10. https://doi.org/[10.1007/s11469-020-00277-1](https://doi.org/10.1007/s11469-020-00277-1)

Sun, L., Sun, Z., Wu, L., Zhu, Z., Zhang, F., Shang, Z., & Liu, N. (2020). Prevalence and Risk Factors of Acute Posttraumatic Stress Symptoms during the COVID-19 Outbreak in Wuhan, China. *MedRxiv*. <https://doi.org/10.1101/2020.03.06.20032425>

Taylor, S., Landry, C. A., Paluszek, M. M., Fergus, T. A., McKay, D., & Asmundson, G. J. G. (2020). Development and initial validation of the COVID Stress Scales. *Journal of Anxiety Disorders*, *72*, 102232. https://doi.org/10.1016/j.janxdis.2020.102232

Tull, M. T., Barbano, A. C., Scamaldo, K. M., Richmond, J. R., Edmonds, K. A., Rose, J. P., & Gratz, K. L. (2020). The prospective influence of COVID-19 affective risk assessments and intolerance of uncertainty on later dimensions of health anxiety. *Journal of Anxiety Disorders*, *75*, 102290. https://doi.org/10.1016/j.janxdis.2020.102290

Tyra, A. T., Griffin, S. M., Fergus, T. A., & Ginty, A. T. (2021). Individual differences in emotion regulation prospectively predict early COVID-19 related acute stress. *Journal of Anxiety Disorders*, *81*, 102411. https://doi.org/10.1016/j.janxdis.2021

Wang, C., Pan, R., Wan, X., Tan, Y., Xu, L., Ho, C. S., & Ho, R. C. (2020). Immediate psychological responses and associated factors during the initial stage of the 2019 coronavirus disease (COVID-19) epidemic among the general population in China. *International Journal of Environmental Research and Public Health, 17,* 1729. https://doi.org/10.3390/ijerph17051729.

Xiong, J., Lipsitz, O., Nasri, F., Lui, L. M., Gill, H., Phan, L., ... & McIntyre, R. S. (2020). Impact of COVID-19 pandemic on mental health in the general population: A systematic review. *Journal of Affective Disorders*, *277*, 55–64. https://doi.org/10.1016/j.jad.2020.08.001

Xu, C., Xu, Y., Xu, S., Zhang, Q., Liu, X., Shao, Y., Xu, X., Peng, L., & Li, M. (2020). Cognitive reappraisal and the association between perceived stress and anxiety symptoms in COVID-19 isolated people. *Frontiers in Psychiatry*, *11*, 85[8. https://](file:///C:\Users\salvo\Downloads\8.%20https:\)doi.org/10.3389/fpsyt.2020.00858
